# Early Identification of Mobility Limitations in Community-Dwelling Middle-Aged and Older Adults: Development of a Prediction Model Based on a Prospective Cohort

**DOI:** 10.2196/77187

**Published:** 2026-05-11

**Authors:** Alberto Conde Freniche, Wei Hu, Mo Chen, Dantong Wang, Denis Breuillé, Yu-ming Chen

**Affiliations:** 1 Nestlé Institute of Health Sciences, Nestlé Research, Société des Produits Nestlé, Lausanne, Switzerland; 2 Department of Epidemiology, School of Public Health, Sun Yat-sen University, No. 135, Xingang Xi Road, Guangzhou, China, 86 20-87330605

**Keywords:** prediction model, prognostic, China, self-assessment, mobility, mobility limitations, sarcopenia, frailty, diet, machine learning, healthy aging

## Abstract

**Background:**

With the aging of the global population, preventing the onset of mobility limitations is considered a worldwide public health priority.

**Objective:**

This study aimed to develop a predictive model for incident early mobility limitations (EMLs) in late middle-aged and older adults, based on a simple functional test and modifiable lifestyle factors to facilitate a home-based self-assessment of early mobility decline and promote lifestyle intervention strategies.

**Methods:**

Our study population was community dwellers aged 45 years and above who participated in the second and fourth waves of the Guangzhou Nutrition and Health Study. The included participants were healthy, nonfrail adults reporting no limitations in activities of daily living at baseline. At the 6-year follow-up, participants with poor physical performance (walking speed <1 m/s or handgrip strength <28 kg for males and <18 kg for females) or reporting some difficulty in walking and/or climbing stairs were classified as experiencing EMLs. Least absolute shrinkage and selection operator (LASSO) was used to identify predictors from various factors, and 6 machine learning models were trained and evaluated for EML prediction, using bootstrap-based techniques to address class imbalance. Predictive ability was quantified using the area under the receiver operating characteristic curve (AUC). Variable importance analysis was used to identify key predictors.

**Results:**

A total of 1344 participants were included in the analysis, of which 206 (15.33%) developed EMLs after a median follow-up of 6.67 (IQR 5.91‐7.26) years. Those who developed EMLs were older, had a higher BMI, a lower alternate Mediterranean diet score, and poorer performance in the sit-to-stand (STS) test, as well as lower estimated muscle power from the STS at baseline. The final models included 6 out of 9 predictors: age, sex, BMI, alternate Mediterranean diet score, STS power, and dietary calcium intake. Four machine learning models (logistic regression, LASSO, support vector machine, and neural network) achieved an acceptable AUC value (≥0.70) or slightly below in the testing dataset, with the neural network performing the best (AUC 0.70, 95% CI 0.63‐0.77). Curve analysis showed positive net benefits for LASSO and logistic regression. Bootstrapping did not improve classification performance. Advanced age, lower adherence to Mediterranean diet, lower muscle power estimated from STS test, and higher BMI at baseline emerged as the most important predictors of EMLs.

**Conclusions:**

This study shows that EMLs in Chinese adults aged 45 years and older can be predicted using easy-to-obtain physical performance measures, age, sex, BMI, and specific nutritional factors. The combination of prediction models and variable importance analysis provides valuable insights for the early identification and intervention of EMLs. More efforts are needed to validate our findings in external cohorts.

## Introduction

Maintaining optimal locomotive capacity—in this study referred to as “mobility”—is essential to successful aging. Optimal mobility determines the capacity to perform activities of daily living (ADLs) and engage in social and leisure activities. However, aging has a negative impact on mobility, underpinned by multiple mechanisms affecting the musculoskeletal, cardiorespiratory, and neurological systems. Along with the onset of chronic conditions, the aging process increases the likelihood of developing mobility limitations, such as difficulties in running, walking, or climbing stairs. These limitations often cause older adults to lose independence, reducing their quality of life and dramatically increasing health care costs. Therefore, with the aging of the global population, preventing the onset of mobility limitations is considered a worldwide public health priority [1,2].

Mobility limitations are often the first signs of further functional decline. The interplay between the emergence of mobility limitations, sarcopenia, and physical frailty involves intricate relationships. Sarcopenia, defined as “a progressive and generalized loss of skeletal muscle mass and strength” [3,4], requires the presence of at least 1 mobility limitation for its diagnosis, assessed through objective physical performance tests. In turn, sarcopenia is an important component of the physical frailty syndrome [5], where mobility limitations make up 2 out of 5 total diagnostic criteria, together with weight loss, low energy expenditure, and exhaustion [6]. The development of mobility limitations is therefore a critical component at the intersection of sarcopenia and physical frailty, serving as a primary target for interventions. In accordance, the World Health Organization has recently called health care professionals and gerontologists to focus on strategies for maximizing and preserving mobility across the entire life course independent of any specific disease.

The early stage of mobility decline is a window of opportunity for interventions, such as adequate nutrition intake and active exercise, to positively modify the trajectory in later life. Once physical frailty becomes manifest in older adults, it is increasingly challenging to reverse the development of mobility limitations [7]. Notably, the progressive age-related mobility decline may not clinically manifest at early stages because during the aging process, people unconsciously modify the way tasks are performed routinely to compensate for the accumulating physiological deficits [8]. For example, older adults typically increase the step width to preserve their balance while walking [9]. This strategy is maintained until the capacity reserve is exhausted and limitations become manifest. Therefore, to prevent mobility limitations, it is important to identify people who do not yet manifest them but who are at risk for their onset in the near future [10]. A recent scoping review and expert consensus supports the use of the term preclinical or early mobility limitation (EML), referring to such subtle and often unconscious changes in performance [11].

Models to predict EMLs in late middle-aged adults are scarce. Most of the models available were developed to predict mobility disability in older adults based on common physical performance measures, such as gait speed, balance tests, and handgrip strength [12-15]. However, these measures have poor sensitivity to detect mobility deficits in younger adults (under 65 y) [16,17]. Notably, measuring muscle power may increase the sensitivity of prognostic models compared to more commonly used muscle strength measures, such as handgrip strength. Muscle power declines at an earlier and faster rate than muscle strength and is likely to be a more discriminant predictor of mobility limitations [18,19]. Specifically, sit-to-stand (STS) transitions have been shown to decline about 2 decades earlier than handgrip strength and gait speed [16]. Therefore, it is possible that new predictive models built with power-based measures can improve their prognostic value among middle-aged adults. Recently, a formula to calculate muscle power from the STS test has been developed and validated, overcoming the difficulties of evaluating muscle power without any instrument [20,21].

Similarly, the importance of nutrition in preserving mobility has been studied [22,23]. Including nutrition elements in the prediction model would enable a holistic intervention approach to reduce the risk of mobility limitation. To date, few studies have investigated the association between muscle power-based physical performance measures or dietary factors and the development of EML among late middle-aged adults.

In the present study, we aimed to develop and internally validate a model to predict future preclinical mobility limitations in late middle-aged and older adults for patient self-assessment, based on simple–to-obtain measurements, including self-reported data and the STS test, using a cohort of healthy adults aged 45 years and above recruited in a community-based study in the south of China. This model could be used to identify which persons may benefit from preventive strategies at middle and older age to decelerate or delay the onset of mobility limitations in the Chinese population.

## Methods

### Guidelines for Model Development and Validation

We adhered to the TRIPOD (Transparent Reporting of a multivariable prediction model for Individual Prognosis or Diagnosis) guidelines, which provide transparent reporting standards for developing and validating prediction models for individual prognosis or diagnosis [24,25].

### Study Design and Participants

This retrospective cohort study used data from the Guangzhou Nutrition and Health Study (GNHS), which is a longitudinal community-based cohort conducted by the School of Public Health, Sun Yat-sen University, Guangzhou, China. Between 2008 and 2013, a total of 4048 participants aged between 40 and 80 years, who lived in Guangzhou city for at least 5 years, were enrolled in the GNHS and have been followed up every 3 years approximately. Detailed information about the cohort protocol and baseline characteristics can be found elsewhere [26]. Briefly, data were collected from study visits from healthy participants, including physical performance tests, body composition measurements, dietary, and lifestyle factors. A validated semiquantitative Food Frequency Questionnaire was used to collect dietary intake information [27]. Exclusion criteria such as myocardial infarction, stroke, diabetes, cancer, low-energy fractures, mental and physical disability, and chronic liver and kidney diseases were confirmed at the first enrollment.

Participants in the current analysis participated in 2 follow-ups of the GNHS (waves 2 and 4). At wave 2, from October 2013 to July 2017, 2970 participants were asked to take the 5-time STS test. Therefore, this study used wave 2 as the baseline. In addition, participants completed the ADL Scale questionnaire, grip strength test, and 4-minute walking test in wave 2 and subsequent follow-ups. The latest follow-up with available ADL and performance data was selected to allow EMLs to develop. At wave 4, from October 2020 to December 2023, 1952 participants had been followed-up.

### Ethical Considerations

Written informed consent was obtained from all participants. The GNHS was approved by the Ethics Committee of the School of Public Health, Sun Yat-sen University (approval code: SYSU-GWYL2018048; approval date: July 17, 2018) and registered at ClinicalTrials.gov (NCT03179657). All procedures followed were in accordance with the ethical standards of the responsible committee on human experimentation (institutional and national) and with the World Medical Association Declaration of Helsinki. Data were anonymized and were accessible only to the research team. No financial compensation was provided.

### Predicted Outcome

Mobility limitations were assessed using objective mobility tests and perceived difficulties by ADL questionnaire. Participants rated their ease at walking and climbing stairs as independent, have some difficulties, need help, and unable to mobilize. In the predictive model, EML was determined for those individuals meeting at least 1 of the following 3 criteria: (1) some difficulty in walking and/or climbing stairs; (2) poor handgrip strength (handgrip strength <28 kg for males and <18 kg for females); and (3) slow walking speed (<1 m/s).

Validated preclinical thresholds for middle-aged adults were lacking at the time of the study. Gait speed <1 m/s is a common threshold of slow walking speed among well-functioning older adults, and is often treated as a functional benchmark in middle-aged and older adults [28-31]. This threshold is recommended by the Asian Working Group on Sarcopenia (AWGS 2019) to screen for possible sarcopenia and has been applied to cohorts that include midlife participants across Asian countries, including China [32,33]. Similarly, for low handgrip strength, the threshold proposed by the AWGS 2019 was used in this study.

### Baseline Candidate Predictors

The STS test is used to assess functional lower limb strength, transitional movements, balance, and fall risk in older adults. STS test score is based on the amount of time (to the nearest decimal in seconds) a participant is able to transfer from a seated to a standing position and back to sitting 5 times. The equipment needed to perform the STS test includes a stopwatch and standard height chair with straight back (42 cm high). Estimated STS power is calculated based on the results of the STS test, using the subject’s height and BMI and the height of the chair.

In addition to STS power, other prefrailty phenotype-related predictors were considered, such as age, sex, BMI, alternate Mediterranean diet score (aMDS), energy-adjusted daily intake of vitamin D, protein, and calcium (Ca), and the use of vitamin supplements. Age, gender, and vitamin supplement use were self-reported by participants. BMI was measured by physical examination, the details of which are described in our previous study [26]. Nutrient intake levels were estimated based on a semiquantitative Food Frequency Questionnaire. The adherence to the alternate Mediterranean diet was measured by aMDS, calculated by summing the dichotomous points for the items of higher intakes of whole grain, vegetables, fruits, legumes, nuts, fish, and the ratio of monounsaturated fatty acids:saturated fatty acids, lower red meat, and moderate ethanol consumption, as described previously [34].

Finally, a least absolute shrinkage and selection operator (LASSO) regression model was built to screen the predictors for machine learning (ML) model. Feature selection was performed only in the training dataset. After feature selection, 6 variables (age, sex, BMI, aMDS, STS power, and dietary Ca intake) were selected from 9 predictors to construct a prediction model (Figure S1 in Multimedia Appendix 1).

### Statistical Analysis

The continuous measurement data were represented as mean (SD). Student *t* test was used to compare differences between the EML and the normal (no EML) groups. The chi-squared test was used to compare categorical variables. Data were analyzed using R software (version 4.1.0; R Foundation for Statistical Computing).

### ML Stages

The data were preprocessed before the models were trained. The predictors exhibit nonzero variance, and there is no significant correlation between them (Pearson correlation coefficient <0.75). Subsequently, the original dataset was randomly divided into a training dataset with 70% (940/1344) of the sample (796 normal and 144 EMLs) and a testing dataset with 30% (404/1344) of the sample (342 normal and 62 EMLs). Some predictor variables had a percentage of missing data of <10% (Figure S2 in Multimedia Appendix 1). For missing data, the training dataset was imputed with the random forest (RF) method by “mice” package [35]. Missing values of the testing dataset were imputed using median (for numerical) and mode (for categorical), with imputation parameters estimated from the training dataset to avoid leakage. This study performed variable (categorical and numerical) standardization on both the training and testing datasets.

Class imbalance is a common challenge encountered in medical datasets, and it can significantly impact the performance of classification models. The presence of imbalanced classes often leads to overfitting of the majority class data by learning algorithms, resulting in decreased classification performance [36]. Sampling is a widely used technique for adjusting the size of training datasets, and undersampling and oversampling are common approaches [37]. To deal with binary classification problems in the presence of imbalanced classes, synthetic balanced samples were generated according to “ROSE (Random Over-Sampling Examples)” package [38]. ROSE is a bootstrap-based technique that aids the task of binary classification in the presence of rare classes. We combined oversampling and undersampling to rectify the imbalance in data classification. The distribution of the observations by category-wise in the balanced and imbalanced training datasets is shown in Figure S3 in Multimedia Appendix 1.

After the work of preprocessing, the imbalanced and balanced datasets were divided into 5 random parts in the cross-validation test, which was repeated 10 times to randomize the training dataset and improve model performance. In this stage, the models were manually adjusted during training and selected based on the best value of the receiver operating characteristic (ROC), specificity, and sensitivity. The values of these metrics and the parameters used are shown in Table S1 in Multimedia Appendix 1. Calibration curve and decision curve analyses (DCA) were used to evaluate the calibration and clinical utility of the models. The following 6 ML models were trained: logistics regression (LR), RF, support vector machine (SVM), neural network (NNET), lasso regression (LASSO), and Light Gradient-Boosting Machine (LightGBM).

Finally, the testing dataset was used to evaluate the performance of the models trained on the imbalanced and balanced datasets, to assess how well these models fitted the data that was not used during the training stage into the testing dataset. The ROC and precision-recall curves and the metrics area under the receiver operating characteristic curve (AUC), *F*_1_-score, accuracy, balanced accuracy, specificity, sensitivity, positive predictive value (PPV), and negative predictive value were used to assess the performance of the 6 models. The Youden index was used to determine the optimal threshold for the prediction of EML by optimizing specificity and sensitivity. The “tidymodels” package was used in the ML stages.

The Shapley Additive Explanations (SHAP) analysis was used to interpret the model and assess the marginal contribution of each feature to the model output. The waterfall of individualized SHAP was used to interpret single-sample data, illustrating the direction and strength of influence that an individual’s variable values have on the predictive outcome.

## Results

### Population Characteristics

**Figure 1.**
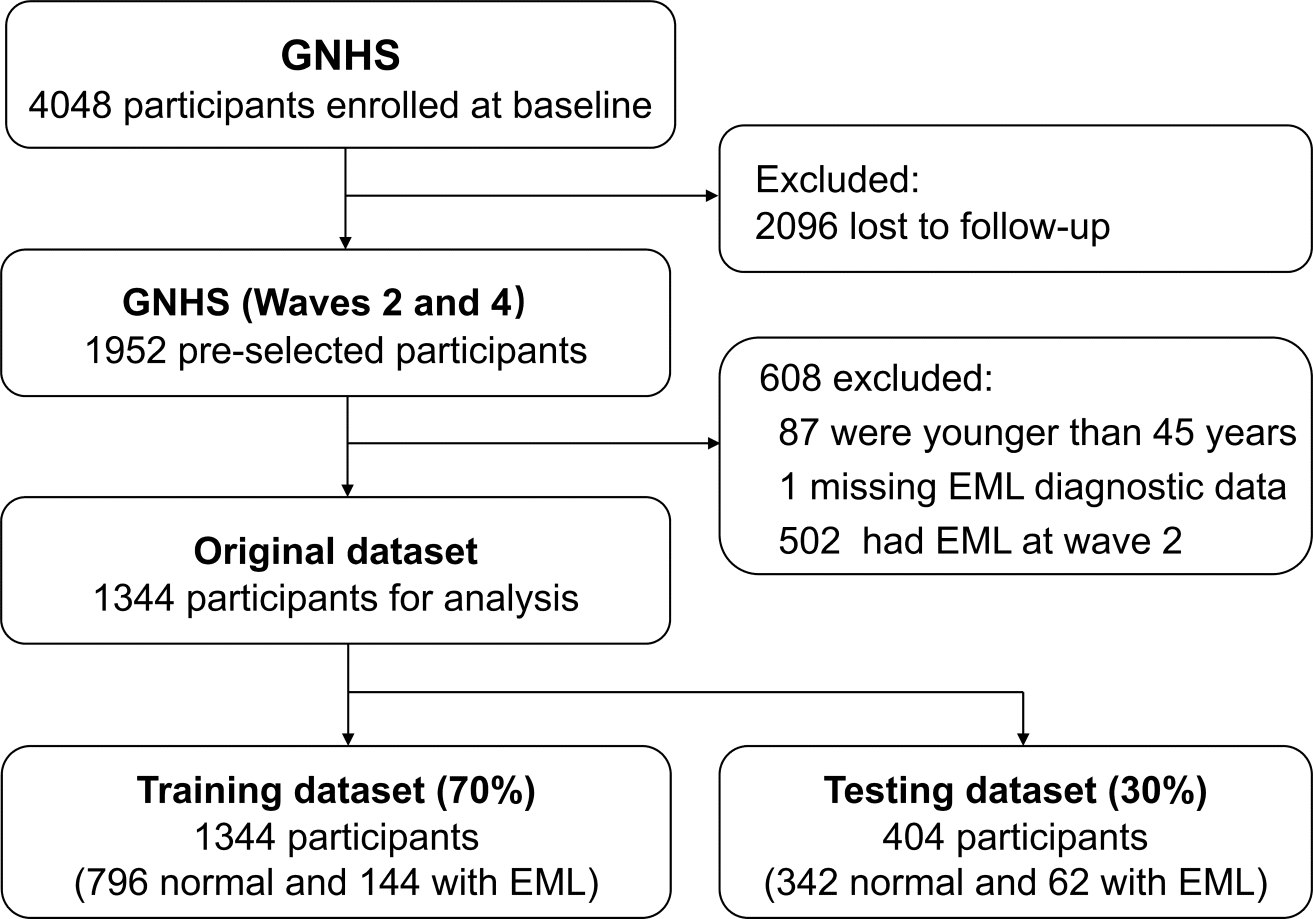
Flowchart of the machine learning stages in this study. EML: early mobility limitation; GNHS: Guangzhou Nutrition and Health Study.

In the final analysis, 1344 participants attended the follow-up and were included. At baseline, the median age was 61.99 (IQR 59.03‐65.68) years, and most participants were women (945/1344, 70.3%). After a median follow-up of 6.67 (IQR 5.91‐7.26) years, 15.33% (206/1344) participants developed EMLs at 6-year follow-up (Figure 1). Those who developed EMLs were older, had a higher BMI, a lower aMDS, and poorer performance in the STS test, as well as lower estimated muscle power from the test (STS power) at baseline (Table 1).

**Table 1.** Baseline candidate variables selected for machine learning by mobility limitations at follow-up in the dataset.^a^^,b,c,d^

Baseline variables	Preclinical mobility limitations (EML^e^)			*P* value
	Total (N=1344)	No EML (n=1138)	EML (n=206)	
Age, y, mean (SD)	62.56 (5.14)	62.21 (5.03)	64.46 (5.33)	<.001
Sex, n (%)				.29
Female	945 (70.31)	807 (70.91)	138 (66.99)	
Male	399 (29.69)	331 (29.09)	68 (33.01)	
BMI (kg/m^2^), mean (SD)	23.38 (3.17)	23.30 (2.99)	23.84 (4.02)	.02
Protein (g/d), mean (SD)	69.07 (10.50)	69.15 (10.54)	68.66 (10.31)	.54
Vitamin D (IU/d), mean (SD)	83.11 (57.96)	84.03 (59.89)	78.07 (45.62)	.17
Ca (mg/d), mean (SD)	547.46 (164.82)	548.81 (158.83)	540.05 (194.89)	.48
aMDS^f^, mean (SD)	4.11 (1.71)	4.15 (1.71)	3.86 (1.69)	.02
Vitamin supplementation, n (%)				>.99
No	1117 (83.11)	946 (83.13)	171 (83.01)	
Yes	227 (16.89)	192 (16.87)	35 (16.99)	
STS^g^ power (W), mean (SD)	268.41 (92.38)	271.66 (94.40)	250.49 (78.12)	.002
STS (s), mean (SD)	7.68 (1.88)	7.62 (1.92)	8.01 (1.67)	.003

a Continuous variables are presented as mean (SD), and categorical variables are presented as n (%).

b Student *t* tests were used to compare the mean differences between no EML and EML groups.

c Chi-square tests were used to count data.

d Statistics after imputation of missing data.

e EML: early mobility limitation.

f aMDS: alternate Mediterranean diet score.

g STS: sit-to-stand test.

### Models’ Performance

**Figure 2.**
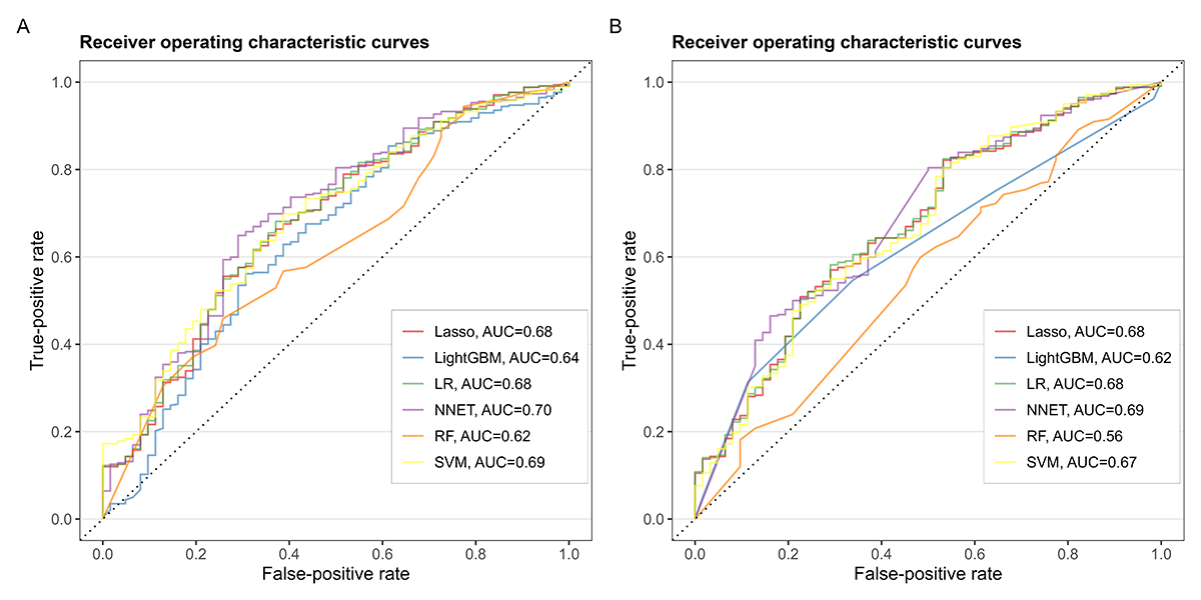
Receiver operating characteristic curves of the models trained with imbalanced (A) and balanced (B) datasets. AUC: area under the receiver operating characteristic curve; LASSO: Lasso regression; LightGBM: Light Gradient-Boosting Machine; LR: logistic regression; NNET: neural network; RF: random forest; SVM: support vector machine.

**Figure 3.**
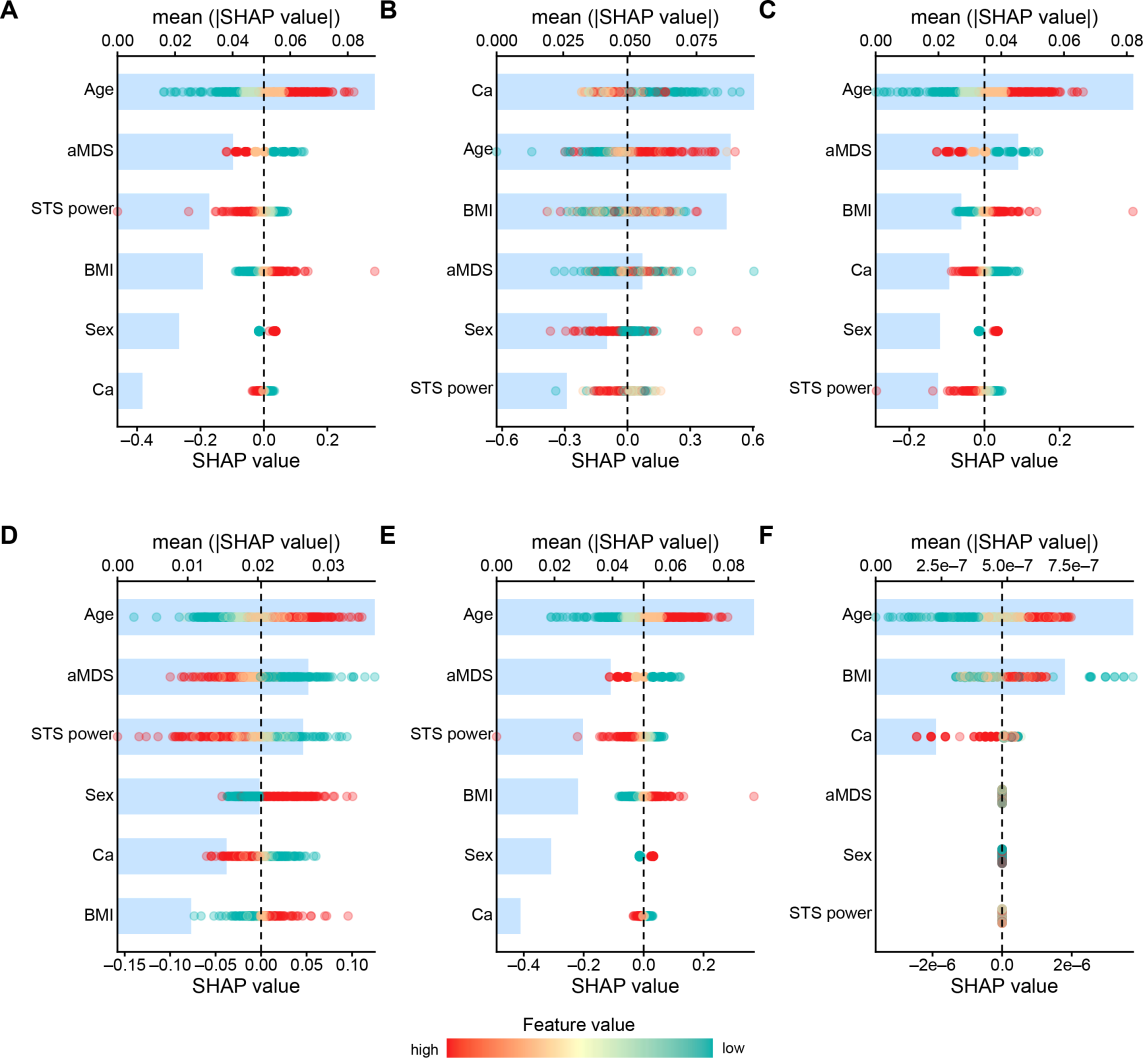
The SHAP for feature importance of the models trained with balanced datasets: (A) logistic regression; (B) random forest; (C) support vector machine; (D) neural network; (E) least absolute shrinkage and selection operator regression; and (F) Light Gradient-Boosting Machine. aMDS: alternate Mediterranean diet score; SHAP: Shapley Additive Explanations; STS: sit-to-stand test.

Figure 2 shows the AUC values for the 6 ML models trained with imbalanced (AUC range 0.62-0.70; Figure 2A) and balanced (AUC range 0.56-0.69; Figure 2B) datasets. All 6 ML models selected all 6 variables (age, sex, BMI, aMDS, STS power, and dietary calcium intake) with different importance (Figure 3), except the LightGBM model for which age, BMI, and dietary calcium intake were not selected. The performance of most models on unseen data remained stable or decreased when the balanced dataset was used, and the LR model maintained the best performance across both datasets.

Table 2 shows the performance of the ML models on unseen data, trained with imbalanced and balanced datasets, based on different metrics. Some models (RF, SVM, and NNET) yielded better values for metric accuracy when they were trained with balanced data, whereas the unbalanced dataset showed better performance in all the other metrics and the rest of the models. LR, NNET, SVM, and LASSO had acceptable values for AUC (≥0.70) or slightly below when trained with imbalanced data, among which NNET had the best values for AUC (0.70; 95% CI 0.63‐0.77) and balanced accuracy (0.68) compared with all the other models trained with imbalanced data. However, NNET models are typically more complex and prone to overfitting. Hence, in conditions of similar performance, simpler models like LR or LASSO may be preferred due to their interpretability and lower risk of overfitting. Specifically, the straightforward coefficients in LR can be easily understood in terms of odds ratios.

**Table 2.** Performance of the models trained with imbalanced and balanced datasets based on different metrics.^a^

Models	Accuracy	Balanced accuracy	*F*_1_-score	PR^a^-AUC^b^	AUC (95% CI)	Threshold	Specificity	Sensitivity	PPV^c^	NPV^d^
Imbalanced dataset										
LR^e^	0.67	0.66	0.92	0.29	0.68 (0.61-0.76)	0.16	0.68	0.63	0.26	0.91
RF^f^	0.50	0.60	0.91	0.23	0.62 (0.55-0.70)	0.08	0.46	0.74	0.20	0.91
SVM^g^	0.69	0.66	0.92	0.27	0.69 (0.62-0.76)	0.15	0.70	0.61	0.27	0.91
NNET^h^	0.66	0.68	0.92	0.29	0.70 (0.63-0.77)	0.32	0.65	0.71	0.27	0.92
LASSO^i^	0.58	0.65	0.92	0.29	0.68 (0.61-0.76)	0.14	0.56	0.74	0.23	0.92
LightGBM^j^	0.58	0.62	0.92	0.23	0.64 (0.56-0.72)	0.18	0.56	0.69	0.22	0.91
Balanced dataset										
LR	0.77	0.64	0.69	0.28	0.68 (0.60-0.75)	0.60	0.82	0.47	0.33	0.90
RF	0.59	0.56	0.74	0.16	0.56 (0.48-0.63)	0.43	0.60	0.52	0.19	0.87
SVM	0.75	0.64	0.71	0.29	0.67 (0.60-0.74)	0.59	0.80	0.47	0.30	0.89
NNET	0.76	0.65	0.66	0.24	0.69 (0.62-0.76)	0.52	0.80	0.50	0.32	0.90
LASSO	0.77	0.64	0.69	0.28	0.68 (0.60-0.75)	0.60	0.82	0.47	0.32	0.90
LightGBM	0.56	0.60	0.92	0.17	0.62(0.55-0.69)	0.50	0.55	0.66	0.21	0.90

a PR: precision-recall.

b AUC: area under the receiver operating characteristic curves.

c PPV: positive predictive value.

d NPV: negative predictive value.

e LR: logistic regression.

f RF: random forest.

g SVM: support vector machine.

h NNET: neural network.

i LASSO: least absolute shrinkage and selection operator.

j LightGBM: Light Gradient-Boosting Machine.

DCA (Figure S8 in Multimedia Appendix 1) results showed that prediction model’s effective range for test datasets are concentrated at low thresholds. All prediction model curves demonstrate positive net benefits exceeding the “no treatment” baseline only within the threshold range of 0% to 50%. When the threshold exceeds 50%, the model’s net benefit drops below zero, losing clinical value.

In calibration curves (Figures S5 and S7 in Multimedia Appendix 1), LASSO regression and logistic regression exhibit relatively good calibration on imbalanced test datasets. However, in balanced datasets, most predictive models show that the predicted probabilities are higher than the actual probabilities, indicating that the models are overconfident in their predictions. For implementation in the real world, prediction models need to be further calibrated according to the specific application scenario.

In addition to discrimination metrics, we assessed model performance using the net reclassification improvement. Logistic regression was selected as reference because it represents a widely used baseline approach for binary risk prediction in preventive settings. Net reclassification improvement was decomposed into event and nonevent components to better understand potential gains and tradeoffs in risk stratification across modeling approaches, and the results are reported separately for models trained on imbalanced and balanced datasets (Table S1 in Multimedia Appendix 1). Only RF achieved a modest improvement in risk reclassification over logistic regression, whereas other ML models did not demonstrate meaningful net reclassification gains.

### Model Interpretability

The SHAP model was used to provide both global and local interpretations of the models. Globally, the more significant contributors to the models trained with the balanced dataset were age, aMDS, STS power, and BMI. Age was among the top 3 most important variables in all 6 models, followed by aMDS in 4 models (LR, SVM, NNET, and LASSO) and STS power (LR, NNET, and LASSO) and BMI (RF, SVM, and light GBM) in 3 models (Figure 3).

**Figure 4.**
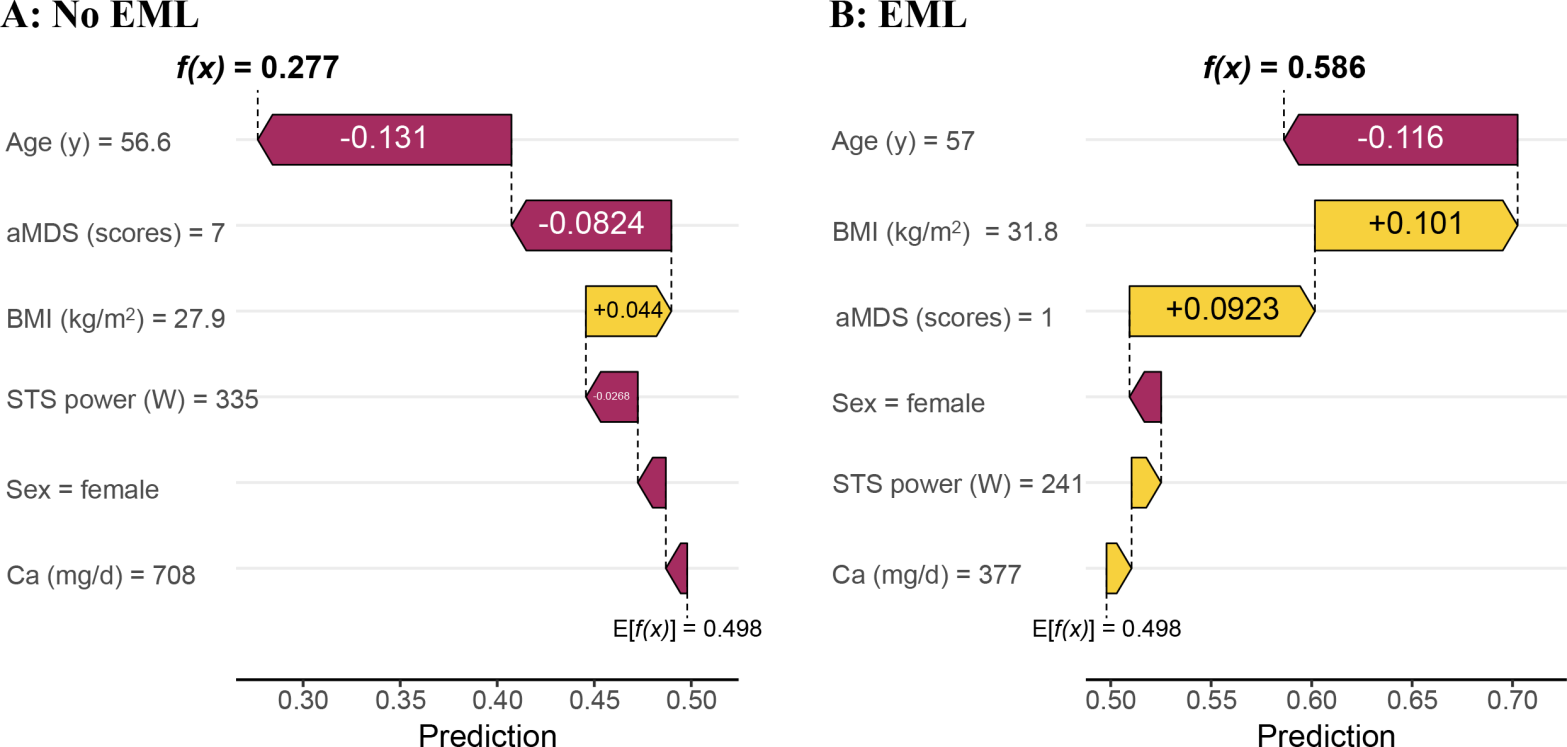
Individualized SHAP waterfall plot for no EML (A) and EML participants (B), demonstrating the personalized contribution of each predictor to the risk score. aMDS: alternate Mediterranean diet score; EML: early mobility limitation; SHAP: Shapley Additive Explanations; STS: sit-to-stand test.

In the single-sample waterfall plot, baseline SHAP value was 0.498, model output values for a randomly selected individual with no EML was 0.277 (Figure 4A), and for an EML individual was 0.586 (Figure 4B). In no EML individuals, lower age as well as higher aMDS scores and STS power were identified as features that had a negative impact on EML, while higher BMI was considered to have a positive impact (Figure 4A). Among EML individuals, lower age was identified as a feature that negatively affected EML, while higher BMI and lower aMDS scores were considered to have a positive impact (Figure 4B).

The SHAP bar plot ranks features by their overall importance, calculated as the mean absolute SHAP value across all participants.

The SHAP beeswarm plot illustrates the impact of each feature on the model’s output for every participant. Each dot represents a participant; its position on the *x*‐axis indicates the SHAP value (positive values increase the prediction of EML), and its color represents the feature’s value (red for high, green for low).

## Discussion

### Principal Results

In this study, we developed 6 prediction models using ML for EML in healthy, mobility-intact middle-aged and older adults from a longitudinal Chinese population-based cohort who were followed-up over 6.67 years. A LASSO regression model was built to select the predictors. Finally, 6 variables (age, sex, BMI, aMDS, STS power, and dietary calcium intake) were identified and included in the model training process. The performance of the different ML models trained with balanced and imbalanced datasets was compared. Advanced age, lower adherence to Mediterranean diet, lower STS power, and higher BMI were identified as the most important predictors of EMLs.

### Comparison to Prior Work

In this study, we have attempted to use commonly used ML models for binary variable prediction, including LR, RF, SVM, NNET, LASSO, and LightGBM. In a recent study with a large sample size (5466), ML models such as SVM, K-nearest neighbor, NNET, RF, and extreme gradient boosting demonstrated good results in predicting frailty among senior people in Canada [39]. Although our database may not have a comparable size, we were interested in exploring the feasibility of applying these ML models to the cohort in Guangzhou, China.

The discrimination power of 4 models (LR, NNET, SVM, and LASSO) trained with the imbalanced data was found acceptable (AUC≥0.70) or slightly below in the testing dataset. The performance moderately decreased when trained with the balanced dataset, with NNET (0.69), LR (0.68), SVM (0.67), and LASSO (0.68) achieving the highest AUC values and, therefore, showing more robust discrimination across datasets (Table 2). Royston et al [40] noted that the AUC of prognostic models is typically between 0.6 and 0.85. The commonly used prognostic tests include the ratio of total plasma cholesterol to high-density lipoprotein cholesterol level, with an AUC of 0.72 for predicting the risk of coronary heart disease [41], and the 6-minute walking test, with an AUC of 0.60 for predicting increased risk of hospitalization among patients with chronic obstructive pulmonary disease [42]. Therefore, the predictive power of our models compares well with that of risk assessment tests widely used in clinical practice that are more invasive (eg, TC/HDL-C from a blood test) or burdensome (eg, 6-minute walking test requiring time and space). Additional DCA of the models trained with imbalanced datasets showed meaningful positive net benefits for LASSO and LR only, supporting the clinical utility of these 2 models within the threshold range of 0% to 35%, but no positive net benefits were found for the balanced trained models (Figure S8 in Multimedia Appendix 1). The incremental predictive benefit of other ML models compared to LR did not demonstrate meaningful net reclassification gains.

Similar ROC-AUC results were obtained by Chaves et al [43], who developed a screening tool from simple–to-obtain self-reported performance measures for predicting the probability of 18-month incident mobility difficulty in community-dwelling women aged 70 to 80 years, with an AUC of 0.73. However, a test is likely to be more sensitive when the prevalence of concomitant risk factors is higher, as it would be expected from older individuals such as those included in that study [44]. Likewise, as the time to event decreases, the predictive accuracy of a model tends to improve [45], leading to less uncertainty in short-term predictions like in the study by Chaves et al [43]. Taken together, considering the challenges of predicting the onset of the early stages of an unspecific and multifactorial condition such as EML in mobility-intact adults who are generally healthy, drawing mostly from modifiable predictors, this study yielded acceptable results for the LASSO and LR imbalanced trained models, with positive net benefits. Given the models’ intended use for early identification of individuals who may benefit from low-risk lifestyle interventions, their limited discrimination remains potentially valuable.

The models developed in this study were designed to maximize clinical applicability. All 6 predictors (age, sex, BMI, STS power, aMDS, and dietary calcium intake) can be easily collected by individuals using a standard chair, a stopwatch, and a short diet questionnaire. These inputs could be incorporated into a digital self-assessment platform or mobile health tool to support interpretation and reporting. Because the predictors include modifiable factors such as diet quality, micronutrient intake, physical function, and body weight, such a tool could help identify individuals at higher risk of mobility decline and provide personalized lifestyle recommendations. The decision threshold can be implemented within preventive care workflows, with thresholds in the range of 10% to 20%—as supported by DCA—offering a balance between early intervention benefits and the likelihood of false positives. This approach may empower individuals to monitor and preserve their mobility health without requiring specialized training or extensive resources.

As expected, age was the most important predictor of incident EML across the different ML models. Advanced age is a risk factor in developing mobility limitations and has been consistently identified as a predictor of mobility outcomes in many predictive equations [46,47]. Therefore, the finding that advanced age was the most important variable across the different ML models as determined by SHAP values (Figure 3) served as a positive control variable in developing our models to predict EML. Other important variables were adherence to the Mediterranean diet, STS power, and BMI. Among the rest of the baseline candidate predictors selected by LASSO regression, other less important variables across the different ML models included being female and lower energy-adjusted calcium intake. Many of these variables confirm some of the risk factors most frequently found to be independently linked to mobility limitations in older people, which include advanced age, higher BMI, and reduced muscle function measured through performance tests [48]. However, our results cast a new light on the predictive ability of estimated muscle power from STS and diet quality.

This study found support for STS power as an important predictor of incident EML in older, as well as in late middle-aged adults. The STS test has been consistently identified as a predictor of subsequent falls, mobility limitations [49], disability [14], osteoporotic hip fractures, and mortality among older adults. Remarkably, we found a higher statistically significant difference and relative difference for STS power (7.79%, *P*=.002) than for STS time values (4.87%, *P*=.003) at baseline between the group developing EML and their counterparts (Table 1). Therefore, only STS power was included among the baseline candidate predictors. Whereas the STS test measures the minimum duration required to rise from a chair a specific number of times (eg, 5-time STS) or the maximum number of STS repetitions within a specified timeframe (eg, 30-s STS), the STS power formula also considers the chair height, and the subject’s body mass and height. Previous evidence in older people suggests that STS power values are more clinically relevant in regard to physical function, cognitive function, and sarcopenia compared to traditional STS measures [20]. Consistent with these findings in older adults, our results extend the predictive value of STS power to identify prospective mobility limitations in late middle-aged adults.

Notably, although normative values for STS power in Asian populations are lacking, the 5-time STS values observed in our study suggest a rather optimal level of physical function in both the EML group (mean 8.01, SD 1.67 s) and the normal group (mean 7.62, SD 1.92 s) at baseline. Specifically, current guidelines from the AWGS 2019 recommend a cut-off of 12 seconds or more to screen for low physical performance and sarcopenia, based on a review of evidence from different Asian cohorts. The striking distance between that cut-off and the baseline functional performance observed in our cohort underlines the ability of our models to identify incident mobility limitations earlier in time, even in the absence of noticeable performance decline. Remarkably, a recent study redefined the cut-off value recommended by the AWGS to 9.2 seconds in men and 10.8 seconds in women aged 60 to 69 years, significantly improving the detection rate of sarcopenia among Chinese adults [50]. These lower thresholds tie well with the values observed in our study and further support the predictive value of subtle deficits in performance tests.

A further novel finding was the importance of diet quality, measured as adherence to the Mediterranean diet, to predict incident EML. Studying the influence of dietary factors on mobility limitation has practical importance, and the findings could provide opportunities for dietary intervention to reduce the potential risk. In this study, we identified adherence to the Mediterranean diet as a predictor of mobility limitations, with a higher adherence score associated with a lower probability of having mobility limitations. This finding is consistent with the previous studies. The aMDS was found positively associated with skeletal muscle mass index, which is an important aspect of mobility, in the Chinese population aged 40 to 75 years [34]. Silva et al [51] conducted a meta-analysis in community-dwelling older people and found that higher adherence to the Mediterranean diet was inversely associated with frailty. The anti-inflammatory and antioxidant properties of some nutrients typically found in higher amounts in Mediterranean diets, such as vitamin C and vitamin E or carotenoids, could contribute to that benefit [52]. Future intervention studies are needed to provide direct evidence on the risk reduction of mobility limitation by adherence to aMDS and improvement of calcium intake among individuals aged 45 years and older.

### Strengths and Limitations

Although many prediction models for mobility limitations or disability have been developed in older people with poorer baseline health and functional performance, to our knowledge, comparable models are lacking for mobility-intact adults under 65 years of age. Therefore, a major strength of the study is the sample selection of healthier and younger participants, allowing for the observation of the early transition from intact mobility to impairment. This design facilitates the identification of early predictors and risk factors of mobility limitations, which might be overlooked in studies restricted to older or already impaired individuals.

Another positive aspect of the study is the selected set of predictor variables, which can be easily self-assessed at home with a smartphone and comprises several modifiable risk factors such as diet, physical function, and weight. As a result, the information collected for the prognostic test could be used for guiding potential intervention strategies. Finally, despite the common practice among ML researchers of using extensive datasets and training models with numerous predictors, this research has shown the feasibility of applying ML in datasets with a reasonable number of predictors and in cases where the classes are initially unbalanced.

There are some limitations of the study. First, the composite outcome EML measured in this research integrated both patient-reported and performance-based measures, as recommended by Richardson et al [11]. The expert group conceptualized the term *early* or *preclinical* mobility limitations to capture subtle decrements in mobility; however, a standardized measure has not yet been established. Further research examining the reliability, validity, and responsiveness of outcome measures is necessary to strengthen the evidence base on preclinical mobility limitation.

Second, the PPV, which indicates the likelihood that a positive test result is a true positive, was not optimal across models. These results should be interpreted considering the relatively low prevalence of EML observed in our cohort (15.3%) compared to that reported in other studies with similar operationalizations of EML among Chinese (27.9%) [53]. Because predictive values are prevalence-dependent, this may have increased the proportion of false positives, lowering the PPVs of our models [54]. This prevalence discrepancy may stem from the high proportion of lost-to–follow-up participants in our study, over 50%. The cohort in this study was followed in a designated location, introducing attrition bias for participants who were more frail, developed disability, or moved to care facilities. To address this discrepancy, we recalculated the PPV using the prevalence in the target population (27.9% in China) for the LR, SVM, NNET, and LASSO models. The PPV increases to approximately 43% to 44% indicating a substantially higher probability that individuals classified as high risk will truly experience the outcome when the model is applied in the intended population. In addition, proper calibration of the models may further optimize the PPV [55]. Notably, in the context of rule-in tests, it is crucial to balance the benefits of correctly identifying at-risk individuals against the potential harm of false positives [56]. The prognostic models developed in this study are aimed at promoting healthy lifestyle advice, like increased physical activity and improved nutrition, which are particularly relevant for the intended at-risk population. Therefore, the limited PPVs observed in our sample shall not limit the applicability of some of our models to identify individuals who may benefit the most from low-risk preventive interventions in the target Chinese population. In particular, a consistent net benefit was shown in DCA for SVM and LR across threshold probabilities roughly in the 10% to 35% band.

Another major limitation of the study was the validation of the models with an internal testing dataset. Further studies on independent cohorts need to be undertaken to establish the validity of our findings. Lastly, we suffered from an imbalanced dataset with only 206 subjects developing EML out of the 1344 sample. Similar imbalance problems have been observed in related studies [37], and this imbalance can lead to biased estimates of training performance [36]. This is because algorithms always tend to predict negative outcomes to achieve higher accuracy, which can also bring implementation challenges, such as when the primary interest lies in discovering the predictive results for EML, and biased predictions render the algorithm meaningless. However, in this study, we used the ROSE bootstrap-based technique [38] to remedy the imbalanced data and generate synthetic balanced samples, leading to more reliable and consistent evaluation of our models across 2 datasets.

### Conclusions

This study is the first, to the authors’ knowledge, to develop a prediction model for identifying the onset of EMLs in middle-aged and older adults based exclusively on at-home, simple–to-obtain measurements. The evaluation of model performance on a testing dataset, considering both imbalanced and balanced data training, indicates the potential utility of the ML approach in identifying individuals who developed EMLs over time. LASSO and logistic regression yielded the most meaningful positive net benefits, supporting their potential clinical utility for early identification of individuals who may benefit from low-risk lifestyle interventions, though external validation is still required. Across models, age, adherence to the Mediterranean diet, STS power, and BMI consistently emerged as the most important predictors. These models provide an initial foundation for developing a digital self-assessment tool to identify individuals at higher risk of mobility decline and support personalized preventive strategies in clinical and at-home settings.

## Supplementary material

10.2196/77187 Multimedia Appendix 1 Supplementary figures and tables.

## Abbreviations

ADL: activities of daily living
aMDS: alternate Mediterranean diet score
AUC: area under the receiver operating characteristic curve
DCA: decision curve analysis
EML: early mobility limitation
GNHS: Guangzhou Nutrition and Health Study
LASSO: least absolute shrinkage and selection operator
LightGBM: Light Gradient-Boosting Machine
LR: logistic regression
ML: machine learning
NNET: neural network
PPV: positive predictive value
RF: random forest
ROC: receiver operating characteristic
ROSE: Random Over-Sampling Examples
SHAP: Shapley Additive Explanations
STS: sit-to-stand
SVM: support vector machine
TRIPOD: Transparent Reporting of a multivariable prediction model for Individual Prognosis or Diagnosis
