# Supplementary material for: Early Identification of Mobility Limitations in Community-Dwelling Middle-Aged and Older Adults: Development of a Prediction Model Based on a Prospective Cohort
Source: JMIR Aging. 2026 May 11;9:e77187. doi: 10.2196/77187 (PMC13160483; doi:10.2196/77187)
Supplement: Multimedia Appendix 1 [file aging-v9-e77187-s001.docx]

## Multimedia Appendix


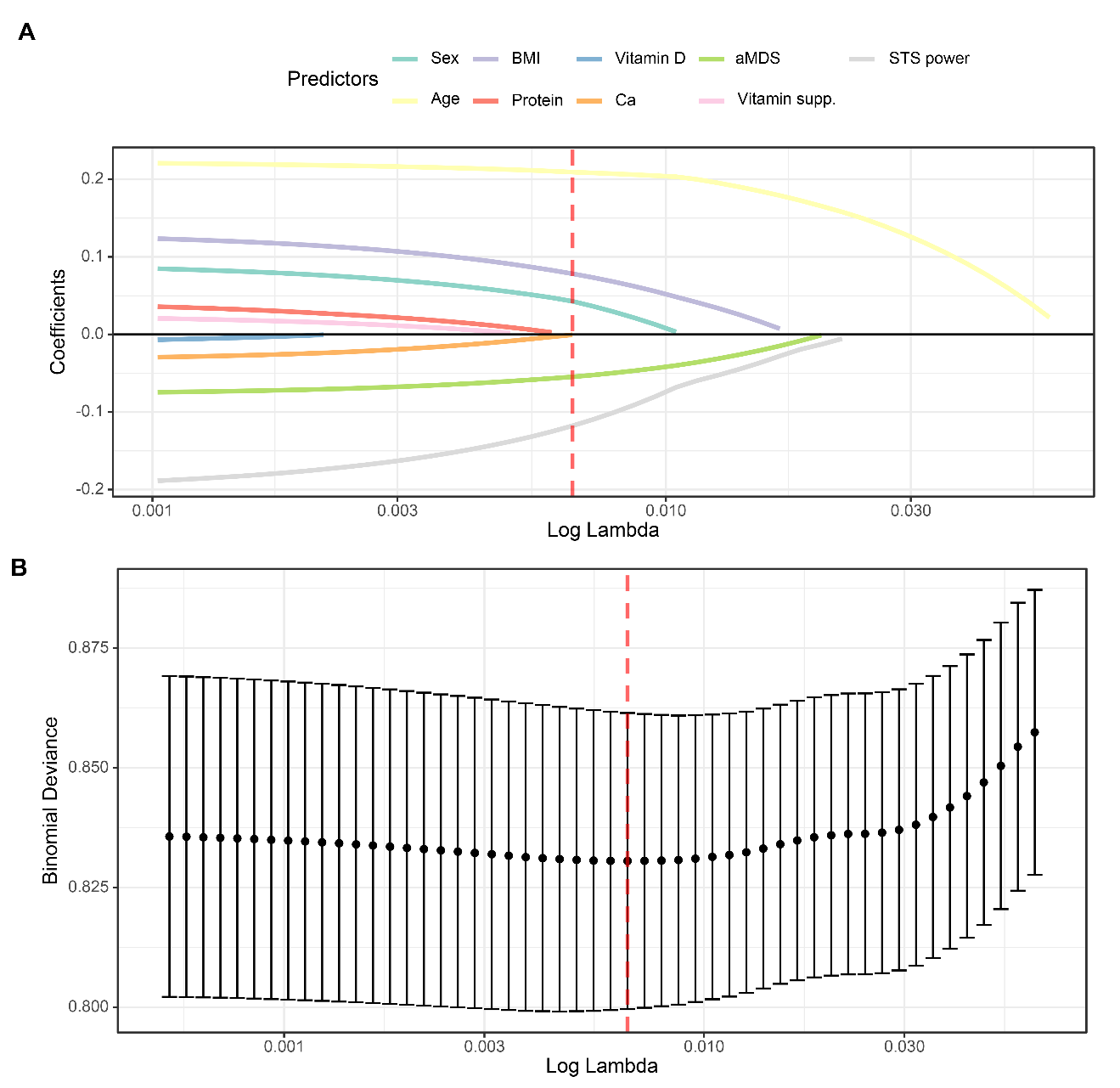


Figure S1. Predictor screening based on lasso regression


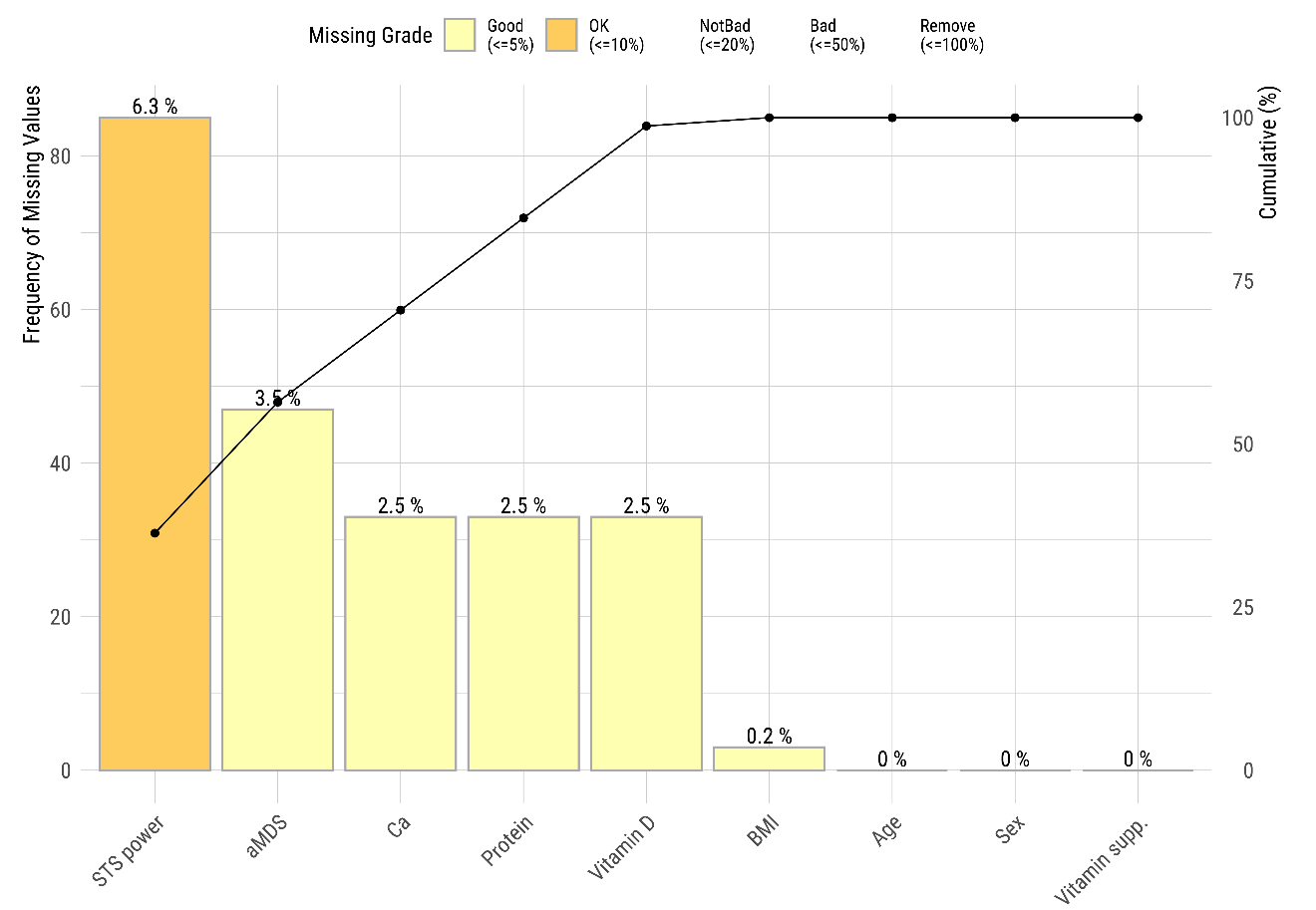


Figure S2. Pareto chart with missing values


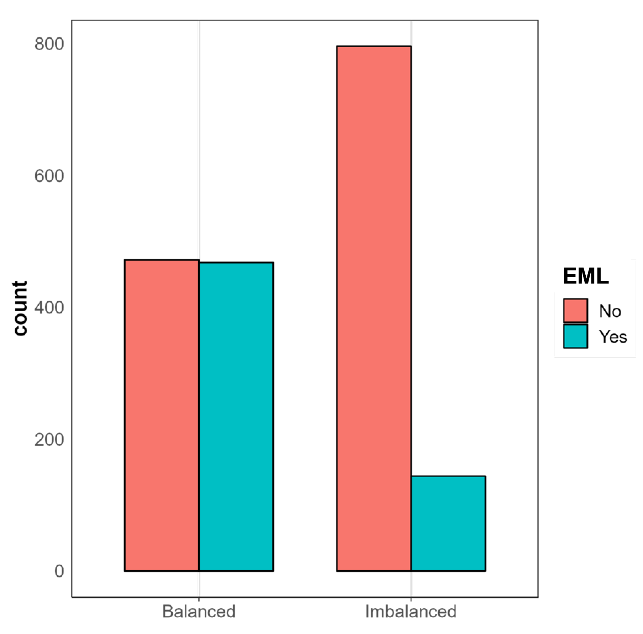


Figure S3. Number of observations in each of the categories of the EML status in the imbalanced and balanced training datasets.


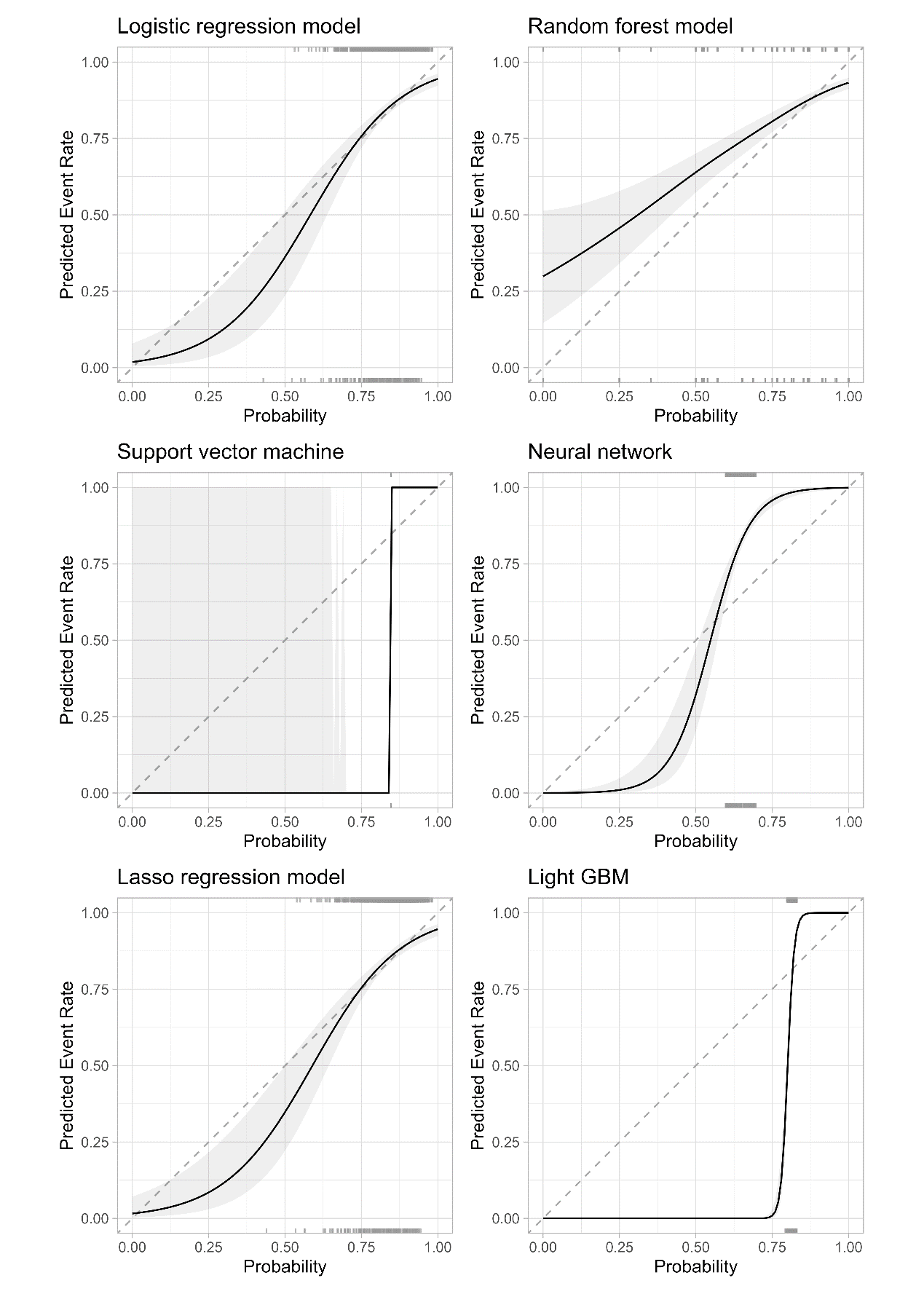


Figure S4 Calibration curve of the models trained with imbalanced datasets for train set.


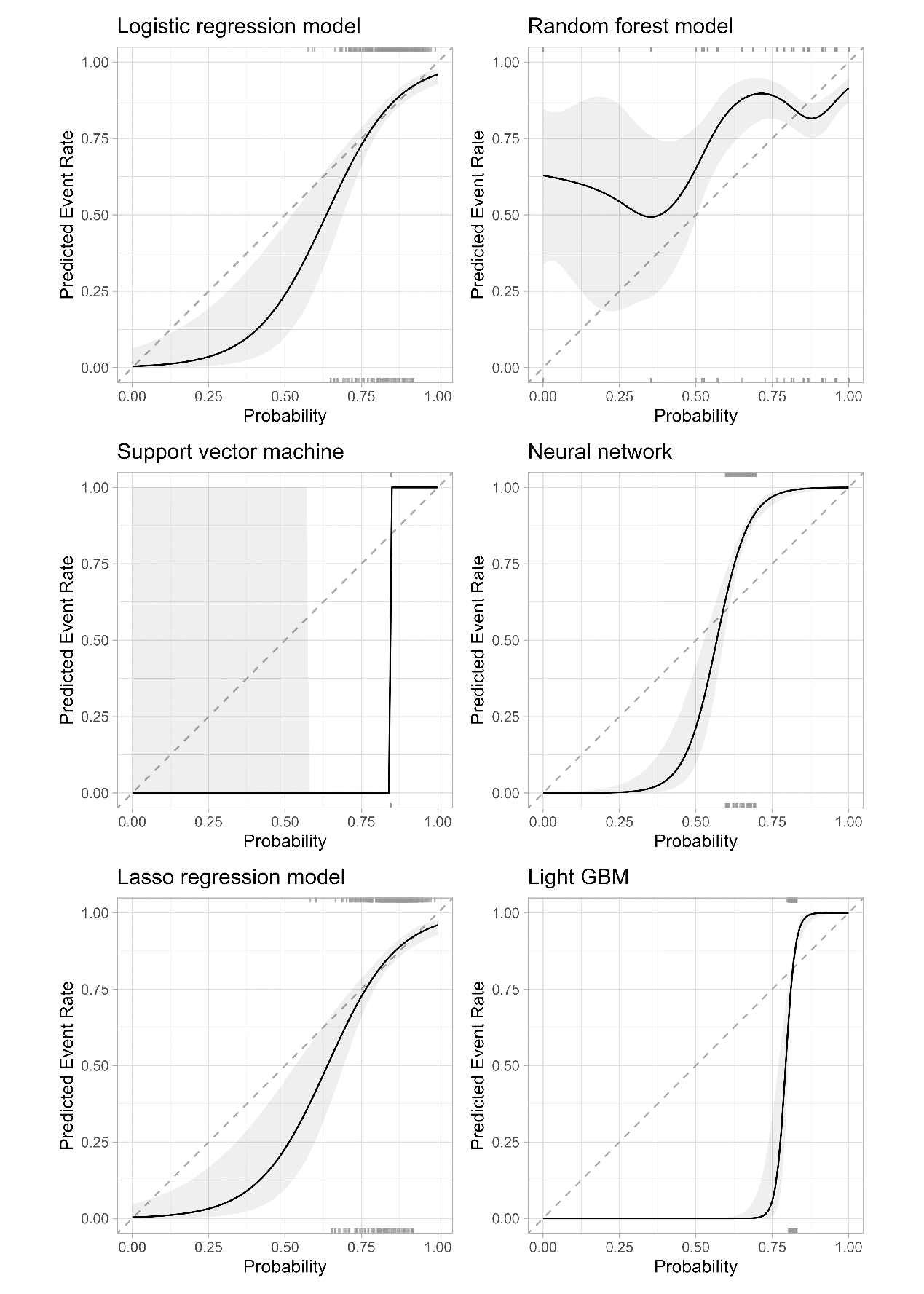


Figure S5 Calibration curve of the models trained with imbalanced datasets for test set.


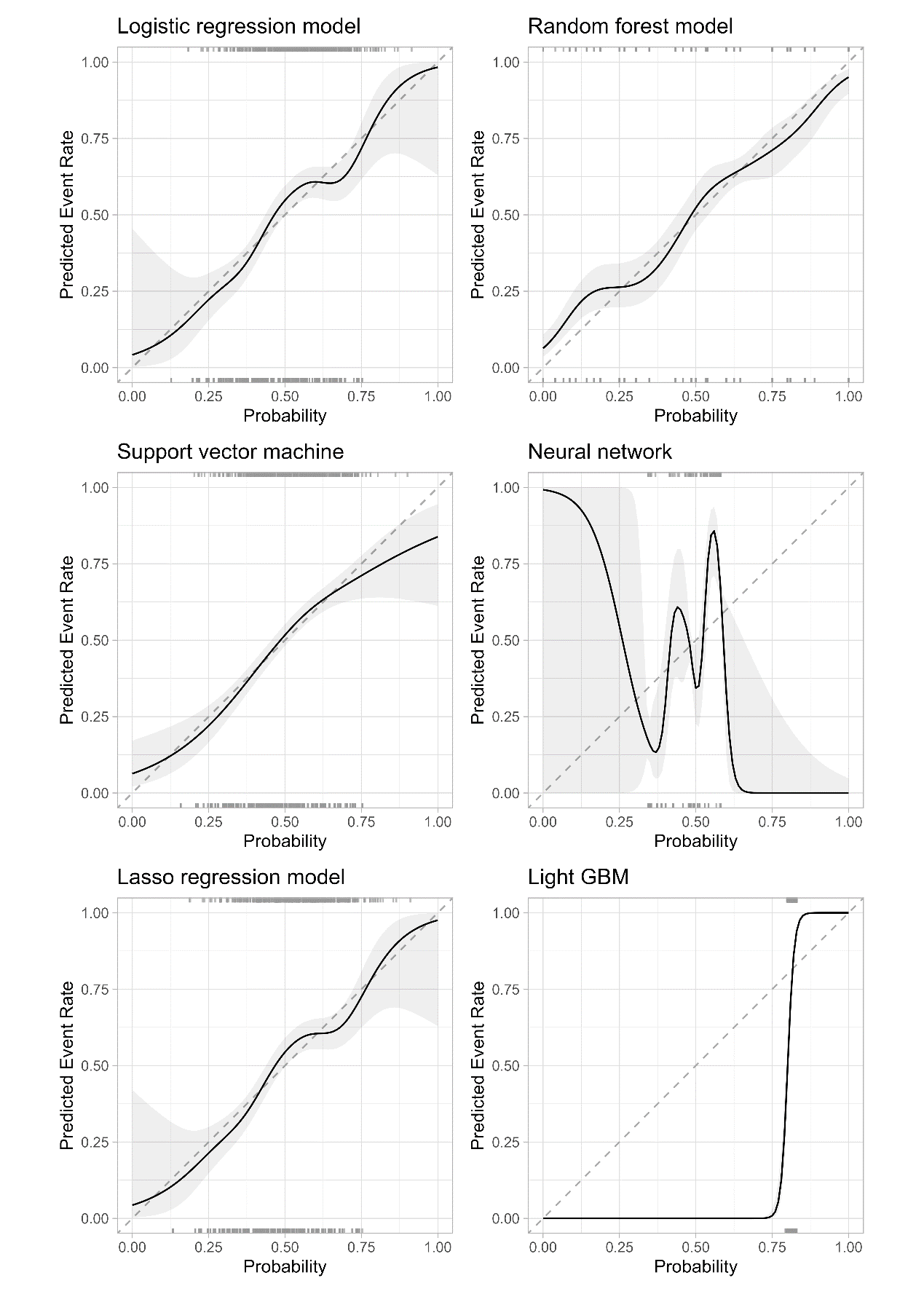


Figure S6 Calibration curve of the models trained with balanced datasets for train set.


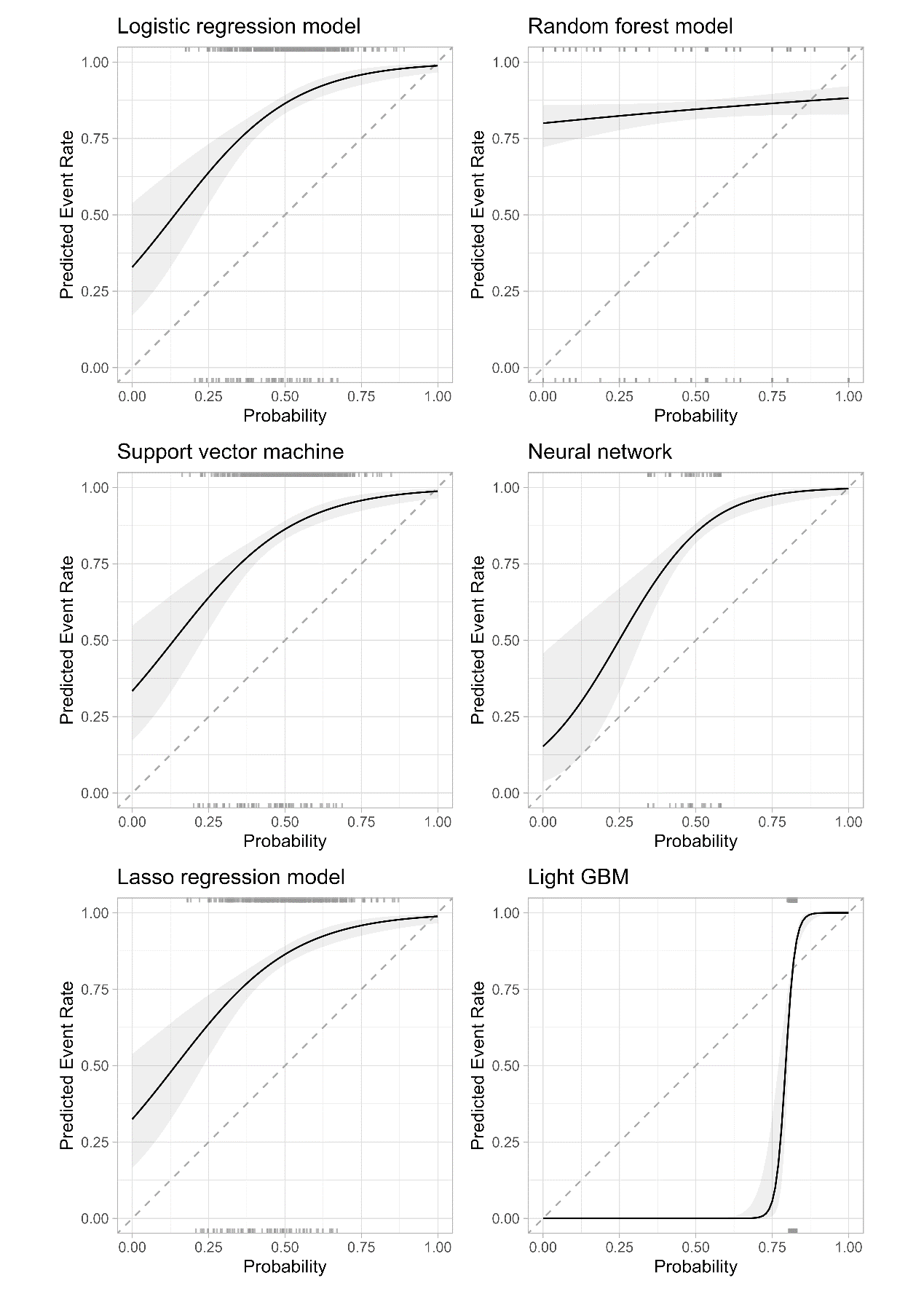


Figure S7 Calibration curve of the models trained with balanced datasets for test set.


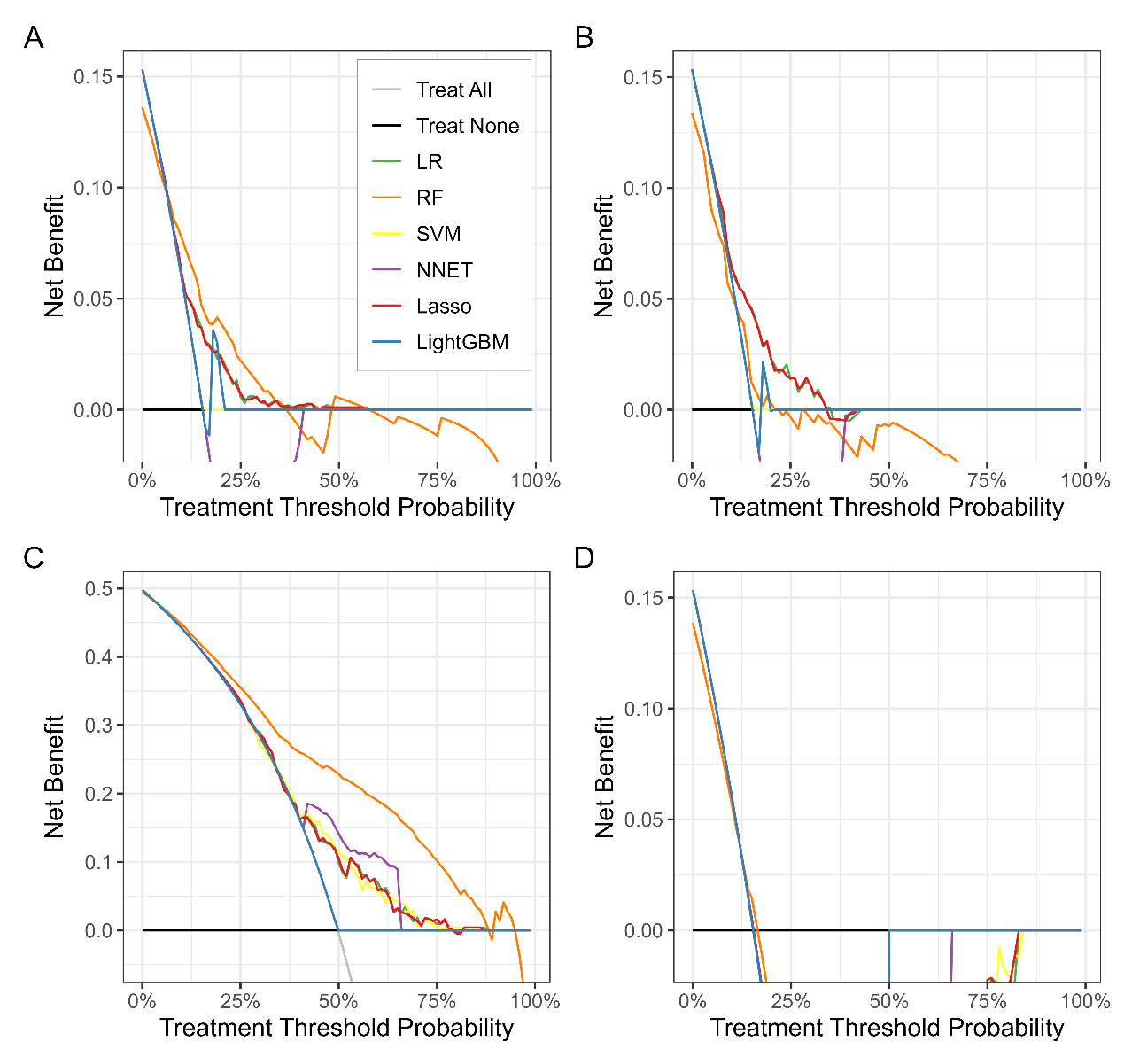


Figure S8 Decision curve analysis of the models trained with imbalanced datasets for train set (A) and test set (B), with balanced datasets for train set (C) and test set (D). Lasso = Lasso regression; LightGBM = Light Gradient Boosting Machine; LR = Logistic Regression; NNET = Neural Network; RF = Random Forest; SVM = Support Vector Machine.


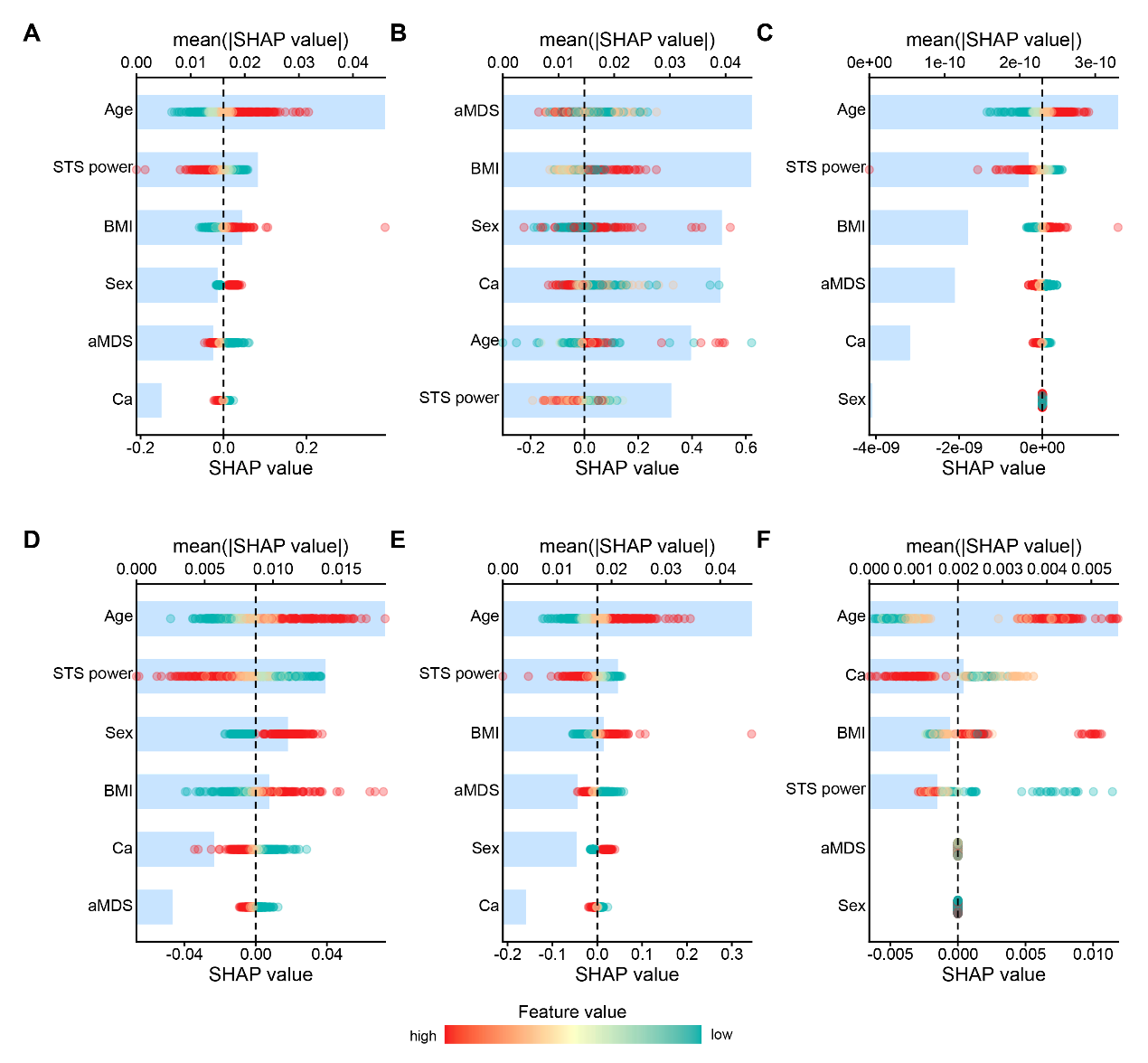


Figure S9 Shapley additive explanation (SHAP) for feature importance of the models trained with imbalanced datasets. (A) LR = Logistic Regression; (B) RF = Random Forest; (C) SVM = Support Vector Machine; (D) NNET = Neural Network; (E) Lasso = Lasso regression; (F) LightGBM = Light Gradient Boosting Machine.

The SHAP bar plot ranks features by their overall importance, calculated as the mean absolute SHAP value across all participants.

The SHAP beeswarm plot illustrates the impact of each feature on the model's output for every participant. Each dot represents a participant; its position on the x‐axis indicates the SHAP value (positive values increase the prediction of EML), and its color represents the feature's value (red for high, green for low).

Table S1 Values of ROC, specificity and sensitivity achieved with each model selected for testing and parameters used

| **Models** | **AUC (95% CI)** | **Specificity** | **Sensitivity** | **Parameters** |
| --- | --- | --- | --- | --- |
| **Imbalanced dataset** | |  |  |  |
| LR | 0.652(0.602,0.702) | 0.619 | 0.632 | - |
| RF | 0.717(0.671,0.764) | 0.796 | 0.535 | mtry=1; trees=1; min_n=40 |
| SVM | 0.646(0.596,0.696) | 0.717 | 0.521 | sigma = 4.478476369061654e-8; Cost = 0.4245695352348959 |
| NNET | 0.655(0.605,0.705) | 0.652 | 0.625 | hidden_units = 1; penalty = 0.01378279233758496 |
| Lasso | 0.651(0.601,0.701) | 0.616 | 0.632 | penalty= 1.014489913051037e-10 |
| LightGBM | 0.712(0.666,0.759) | 0.588 | 0.743 | mtry=2; trees=859; tree_depth=5; loss_reduction= 14.04748988570621; learn_rate= 0.000085718175360686 |
| **Balanced dataset** | |  |  |  |
| LR | 0.649(0.614,0.684) | 0.750 | 0.521 | - |
| RF | 0.796(0.768,0.823) | 0.786 | 0.675 | mtry=1; trees=1; min_n=33 |
| SVM | 0.651(0.616,0.686) | 0.693 | 0.562 | sigma = 0.001226975632767091; Cost = 6.745726500572633 |
| NNET | 0.665(0.632,0.699) | 0.623 | 0.684 | hidden_units = 2; penalty = 0.0004822102471437359 |
| Lasso | 0.649(0.614,0.684) | 0.750 | 0.515 | penalty= 0.001264223949959095 |
| LightGBM | 0.684(0.652,0.717) | 0.665 | 0.641 | mtry=6; trees=3; tree_depth=5; loss_reduction= 11.67152941772497; learn_rate= 0.000003004314568498968 |

Table S2 NRI of the models trained with imbalanced datasets

| **Models** | **NRI** | **Event NRI** | **Non-event NRI** |
| --- | --- | --- | --- |
| **Train dataset** |  |  |  |
| LR | Reference |  |  |
| RF | 0.19 (0.10,0.28) | 0.29 (0.21,0.38) | -0.10 (-0.13,-0.08) |
| SVM | -0.08 (-0.13,-0.03) | -0.12 (-0.17,-0.07) | 0.04 (0.03,0.05) |
| NNET | -0.08 (-0.13,-0.02) | 0.88 (0.83,0.93) | -0.96 (-0.97,-0.95) |
| Lasso | -0.00 (-0.02,0.01) | -0.01 (-0.02,0.00) | 0.00 (0.00,0.01) |
| LightGBM | -0.08 (-0.13,-0.03) | -0.12 (-0.18,-0.07) | 0.04 (0.03,0.06) |
| **Test dataset** |  |  |  |
| LR | Reference |  |  |
| RF | 0.04 (-0.08,0.16) | 0.15 (0.03,0.26) | -0.11 (-0.14,-0.07) |
| SVM | -0.11 (-0.21,-0.03) | -0.13 (-0.23,-0.05) | 0.02 (0.01,0.04) |
| NNET | -0.11 (-0.20,-0.03) | 0.87 (0.78,0.95) | -0.98 (-0.99,-0.96) |
| Lasso | 0.00 (0.00,0.00) | 0.00 (0.00,0.00) | 0.00 (0.00,0.00) |
| LightGBM | -0.11 (-0.20,-0.02) | -0.13 (-0.22,-0.05) | 0.02 (0.01,0.04) |

Table S3 NRI of the models trained with balanced datasets

| **Models** | **NRI** | **Event NRI** | **Non-event NRI** |
| --- | --- | --- | --- |
| **Train dataset** |  |  |  |
| LR | Reference |  |  |
| RF | 0.49 (0.41,0.57) | 0.35 (0.30,0.41) | 0.13 (0.07,0.19) |
| SVM | -0.08 (-0.11,-0.05) | -0.06 (-0.08,-0.04) | -0.03 (-0.05,-0.00) |
| NNET | -0.15 (-0.20,-0.11) | -0.09 (-0.12,-0.06) | -0.07 (-0.10,-0.03) |
| Lasso | -0.02 (-0.04,-0.01) | -0.01 (-0.02,-0.00) | -0.01 (-0.02,-0.00) |
| LightGBM | -0.15 (-0.20,-0.11) | -0.09 (-0.12,-0.06) | -0.07 (-0.10,-0.03) |
| **Test dataset** |  |  |  |
| LR | Reference |  |  |
| RF | -0.05 (-0.24,0.15) | -0.02 (-0.18,0.16) | -0.03 (-0.10,0.04) |
| SVM | -0.01 (-0.09,0.07) | 0.00 (-0.08,0.08) | -0.01 (-0.03,0.01) |
| NNET | -0.19 (-0.30,-0.10) | -0.18 (-0.28,-0.09) | -0.02 (-0.06,0.02) |
| Lasso | -0.00 (-0.01,0.01) | 0.00 (0.00,0.00) | -0.00 (-0.01,0.01) |
| LightGBM | -0.19 (-0.31,-0.10) | -0.18 (-0.28,-0.08) | -0.02 (-0.05,0.02) |
